# Worldwide integrated assessment on systemic pesticides: Global collapse of the entomofauna: exploring the role of systemic insecticides

**DOI:** 10.1007/s11356-014-3220-1

**Published:** 2014-08-23

**Authors:** Maarten Bijleveld van Lexmond, Jean-Marc Bonmatin, Dave Goulson, Dominique A. Noome

**Affiliations:** 1 Task Force on Systemic Pesticides, Pertuis-du-Sault, 2000 Neuchâtel, Switzerland; 2 Centre National de la Recherche Scientifique, Centre de Biophysique Moléculaire, rue Charles Sadron, 45071 Orléans Cedex 02, France; 3 School of Life Sciences, University of Sussex, Brighton, BN1 9QG UK; 4 Kasungu National Park, c/o Lifupa Conservation Lodge, Private Bag 151, Lilongwe, Malawi

**Keywords:** Neonicotinoids, Fipronil, Insecticides, Ecosystem services, Biodiversity, Non-target organisms

## The appeal of Notre Dame de Londres

In July 2009, a group of entomologists and ornithologists met at Notre Dame de Londres, a small village in the French department of Hérault, as a result of an international enquiry amongst entomologists on the catastrophic decline of insects (and arthropods in general) all over Europe.

They noted that a perceptible and gradual decline of insects, as part of the general impoverishment of the natural environment, had set in from the 1950s onwards. Amongst many others, they recognized as root causes of this decline the intensification of agriculture with its accompanying loss of natural habitats and massive use of pesticides and herbicides, the manifold increase in roads and motorized traffic as well as a continent-wide nocturnal light pollution and nitrogen deposition.

They equally agreed that a further degradation of the situation, a steeper decline in insect populations, had started in the decade 1990–2000. This first began in western Europe, followed by eastern and southern Europe, is nowadays apparent in the scarcity of insects splattered on windscreens of motorcars and squashed against their radiators and is best documented in the decline of butterflies and the global disorders amongst honey bees. They concluded that these phenomena reflected the now general collapse of Europe’s entomofauna.

They also noted that the massive collapse of different species, genera and families of arthropods coincided with the severe decline of populations of different insectivorous bird species up to now considered as “common” such as swallows and starlings.

On the basis of existing studies and numerous observations in the field as well as overwhelming circumstantial evidence, they came to the hypothesis that the new generation of pesticides, the persistent, systemic and neurotoxic neonicotinoids and fipronil, introduced in the early 1990s, are likely to be responsible at least in part for these declines.

They, therefore, issued the Appeal of Notre Dame de Londres under the heading “No Silent Spring again” referring to Rachel Carson’s book “Silent Spring” then published almost half a century ago:

The disappearance of honey bees is only the most visible part of a phenomenon now generalized in all of Western Europe. The brutal and recent collapse of insect populations is the prelude of a massive loss in biodiversity with foreseeable dramatic consequences for natural ecosytems, the human environment and public health.

The systematic use of persistent neurotoxic insecticides in intensive agriculture and horticulture (neonicotinoids such as imidacloprid and thiamethoxam, and fipronil as a phenylpyrazole), which now form an invisible, widespread, toxic haze on land, in water and in the air, is regarded as a principal cause of this collapse observed by entomologists beginning in the middle of the 1990’s and followed by the decline of insectivorous and other bird species by the ornithologists.

For this reason the undersigned raise an alarm and demand a much stricter adherence to the « Precautionary Principle » as enshrined in the E.U. Commission’s Directive 91/414, and defined by UNESCO in 2005 as « When human activities may lead to morally unacceptable harm that is scientifically plausible but uncertain, actions shall be taken to avoid or diminish that harm ».

## The international scientific Task Force on Systemic Pesticides (TFSP)

In response, an international scientific Task Force on Systemic Pesticides of independent scientists was set up shortly afterwards by a Steering Committee of which Maarten Bijleveld van Lexmond (Switzerland), Pierre Goeldlin de Tiefenau (Switzerland), François Ramade (France) and Jeroen van der Sluijs (The Nederlands) were the first members. Over the years, membership grew and today counts 15 nationalities in four continents. The Task Force on Systemic Pesticides (TFSP) advises as a specialist group two IUCN Commissions, the *Commission on Ecosystem Management* and the *Species Survival Commission*. Its work has been noted by the *Subsidiary Body on Scientific, Technical and Technological Advice* under the Convention on Biodiversity (CBD) and was brought to the attention of the Intergovernmental Science-Policy Platform on Biodiversity and Ecosystem Services (IPBES) in the context of the fast-track thematic assessment of pollinators, pollination and food production.

In undertaking the Worldwide Integrated Assessment (WIA), over the course of the last 4 years, the TFSP has examined over 800 scientific peer-reviewed papers published over the past two decades. The TFSP areas of expertise span diverse disciplines, including chemistry, physics, biology, entomology, agronomy, zoology, risk assessment and (eco) toxicology, and this has enabled a truly interdisciplinary evaluation of the evidence, necessary to understand the diverse ramifications of the global use of systemic pesticides on individual organisms, on ecosystems and on ecosystem processes and services.

## The findings of the TFSP-WIA

Neonicotinoids were introduced in the early 1990s and are now the most widely used insecticides in the world. They are neurotoxins, binding to nicotinic acetylcholine receptors (nAChRs) in the central nervous system and causing nervous stimulation at low concentrations but receptor blockage, paralysis and death at higher concentrations. Fipronil is another widely used systemic insecticide that shares many of the properties of neonicotinoids and was introduced around the same time; hence, this compound is also included here. Both neonicotinoids and fipronil exhibit extremely high toxicity to most arthropods and a lower toxicity to vertebrates (although fipronil exhibits high acute toxicity to fish and some bird species). They are relatively water soluble and are readily taken up by plant roots or leaves, so they can be applied in a variety of ways (e.g. foliar spray, soil drench and seed dressing). The predominant use of these chemicals, in terms of the area of land over which they are used, is as a seed dressing, whereby the active ingredient is applied prophylactically to seeds before sowing and is then absorbed by the growing plant and spreads throughout the plant tissues, hence protecting all parts of the crop (Simon-Delso et al. 2014).

A range of concerns have emerged as to the impacts of neonicotinoids and fipronil on the environment (Bonmatin et al. 2014; Pisa et al. 2014; Gibbons et al. 2014; Chagnon et al. 2014; Furlan and Kreutzweiser 2014):

- It has become apparent that neonicotinoids can persist for years in soils and so cause environmental concentrations to build up if regularly used. This is likely to be impacting substantially on soil invertebrates, which as a group perform a vital service in maintaining soil structure and in cycling nutrients. Being water soluble, neonicotinoids leach into ponds, ditches and streams and contaminate groundwater. Contamination of marine environments has been observed but as yet has not been monitored systematically. Concentrations exceeding the LC_50_ for aquatic insects frequently occur in waterways, and much higher concentrations have been found in surface water in arable fields and in adjacent ditches. Waterways with higher neonicotinoid concentrations have been found to have depleted insect abundance and diversity.
- Dust created during drilling of treated seeds is lethal to flying insects and has caused large-scale acute losses of honeybee colonies. When applied as foliar sprays, drift is likely to be highly toxic to non-target insects. Non-crop plants, such as those growing in field margins, hedgerows and near contaminated waterways can become contaminated with neonicotinoids either via dust created during drilling, spray drift or contaminated water. This provides the potential for major impacts on a broad range of non-target herbivorous invertebrates living in farmland.
- Neonicotinoids and fipronil are found in nectar and pollen of treated crops such as maize, oilseed rape and sunflower and also in flowers of wild plants growing in farmland. They have also been detected at much higher concentrations in guttation drops exuded by many crops. In bees, consumption of such contaminated food leads to impaired learning and navigation, raised mortality, increased susceptibility to disease via impaired immune system function and reduced fecundity, and in bumblebees, there is clear evidence for colony-level effects. Studies of other pollinators are lacking. Bees in farmland are simultaneously exposed to some dozens of different agrochemicals, and some act synergistically. The impact of chronic exposure of non-target insects to these chemical cocktails is not addressed by regulatory tests and is very poorly understood.
- Although vertebrates are less susceptible than arthropods, consumption of small numbers of dressed seeds offers a potential route for direct mortality in granivorous birds and mammals, for such birds need to eat only a few spilt seeds to receive a lethal dose. Lower doses lead to a range of symptoms including lethargy, reduced fecundity and impaired immune function. In addition, depletion of invertebrate food supplies is likely to indirectly impact on a broad range of predatory organisms, from arthropods to vertebrates.
- The prophylactic use of broad-spectrum pesticides (as seed dressings) goes against the long-established principles of Integrated Pest Management (IPM) and against new EU directives which make adoption of IPM compulsory. Continual exposure of pests to low concentrations of neonicotinoids is very likely to lead to the evolution of resistance, as has already occurred in several important pest species. Although systemic pesticides can be highly effective at killing pests, there is clear evidence from some farming systems that current neonicotinoid use is unnecessary, providing little or no yield benefit. Agrochemical companies are at present the main source of agronomic advice available for farmers, a situation likely to lead to overuse and inappropriate use of pesticides.

Overall, a compelling body of evidence has accumulated that clearly demonstrates that the wide-scale use of these persistent, water-soluble chemicals is having widespread, chronic impacts upon global biodiversity and is likely to be having major negative effects on ecosystem services such as pollination that are vital to food security and sustainable development. There is an urgent need to reduce the use of these chemicals and to switch to sustainable methods of food production and pest control that do not further reduce global biodiversity and that do not undermine the ecosystem services upon which we all depend (van der Sluijs et al. 2014).

The systemic insecticides, neonicotinoids and fipronil, represent a new chapter in the apparent shortcomings of the regulatory pesticide review and approval process that do not fully consider the risks posed by large-scale applications of broad-spectrum insecticides to ecosystem functioning and services. Our inability to learn from past mistakes is remarkable.
